# Status of the Respiratory Therapy Profession in Ghana: A Cross-Sectional Study

**DOI:** 10.7759/cureus.70718

**Published:** 2024-10-02

**Authors:** Charles Asante, Razaz Shaheen, David Lopez, Dorothy Honny, Clement Laryea, Abdullah Alismail

**Affiliations:** 1 Cardiopulmonary Sciences, Loma Linda University, Loma Linda, USA; 2 Public Health, Loma Linda University, Loma Linda, USA; 3 Respiratory Therapy, University of Ghana, Accra, GHA; 4 Medicine, Military Hospital, Accra, GHA

**Keywords:** africa, education, ghana, respiratory care., respiratory therapy

## Abstract

Introduction and purpose: The respiratory therapy (RT) profession in Ghana is a new and emerging field. Therefore, the purpose of this study was to assess and evaluate the RT profession as a needs assessment to help policymakers and key stakeholders shape the profession in Ghana.

Methods: We conducted a cross-sectional study approved by the Institutional Review Board at Loma Linda University. Participants included RTs, RT students, faculty, physicians, nurses, and midwives, who were recruited via email, WhatsApp, and snowball sampling. Data were collected through an anonymous survey consisting of demographic, professional, and attitudinal questions. We analyzed the data using descriptive statistics and inferential tests, employing IBM Corp. Released 2023. IBM SPSS Statistics for Windows, Version 29.0.2.0 Armonk, NY: IBM Corp.

Results: A total of 195 participants were involved in the study from Ghana, with 83 men (42.6%) and 112 women (57.4%), with an average age of 32.33 (SD ± 8.83) years. A majority (56.4%) held bachelor's degrees, with nurses (35.9%) and physicians (21.9%) forming the largest professional groups. The survey found that 64.2% of participants acknowledged a high demand for RTs, yet interaction with RTs was limited due to their limited availability in the region. Notably, 86.8% were familiar with nebulizers, and 80.6% with Ambu bags. The study also highlighted significant challenges in the profession, including overwhelming workloads (64.3%), lack of recognition (100%), and resource shortages. Asthma emerged as the top respiratory condition treated by RTs. The Net Promoter Score for the respiratory therapy profession, at 26.92, demonstrates a moderately positive response, suggesting an increasing need for enhanced interest among professionals in the field of respiratory therapy in Ghana.

Conclusion: To our knowledge, this was the first needs assessment study that evaluated the field of the RT profession in Ghana. The findings indicate robust support for the necessity, development, and expansion of the RT field in the country. Future prospective studies are recommended to further evaluate the impact and effectiveness of the RT profession on patient care outcomes.

## Introduction

Since its establishment in 1947 in Chicago, USA, respiratory care has been in the limelight to date [1]. This is a result of the exponential growth of the prevalence of respiratory diseases across the world [2,3]. The impact of these diseases (COPD, asthma, pneumonia, COVID-19, etc.) on individual families and society in general can be very devastating [4]. This has necessitated individuals, countries, and world bodies to find urgent solutions to mitigate the surge in respiratory diseases. When looking at Africa, newborns have both inadequate and inequitable distribution of proven life-saving neonatal interventions who suffer from respiratory diseases [5]. The WHO in 2004 conducted research into the respiratory conditions in nine African countries (Kenya, Côte d’Ivoire, Morocco, Guinea, South Africa, Botswana, etc.) in an attempt to evaluate the impact of these chronic respiratory diseases (CRD) on Africa. The results showed that respiratory diseases accounted for 19% of total deaths and 15% of disability among the population [6]. Obaseki et al. (2015) assessed the quality, capacity, and resources available for respiratory care in Nigeria and discovered that there were remarkable differences in the quality and capacity for respiratory care in Nigeria [7]. In Ghana, several studies have evaluated and reported the increase in respiratory diseases in the country, such as asthma and pneumonia, upper respiratory tract infections, and acute respiratory infections [8-11]. This shows the need for a profession that is specialized in treating such diseases.

The recent COVID-19 pandemic that took the world as a tsunami exposed the world’s weakness and unpreparedness in dealing with these respiratory diseases. In response to these challenges and to be proactive and have better preparedness in managing and treating respiratory care diseases, interventions have been deployed by many countries and the World Health Organization (WHO) [12-15]. More specifically, the shortage of respiratory therapists made headlines in almost all countries [16-19]. Ghana has taken the initiative and bold decision to start degree programs in RT. Ghana’s premier university, the University of Ghana, Legon, was the first to establish an RT program in Africa [20].

Therefore, the purpose of this study was to 1) assess and evaluate the status of the respiratory therapy profession in Ghana, and 2) evaluate the challenges and barriers faced by the current RTs in Ghana. Lastly, identify any areas of improvement and make recommendations to policymakers and stakeholders within the country and beyond.

## Materials and methods

The study received ethical approval from the Institutional Review Board at Loma Linda University (approval number 5230311) and from the Christian Health Association of Ghana Research Department, Institutional Review Board (IRB: CHAG-IRB05052023). This was a cross-sectional, anonymous survey study. The study was conducted across various healthcare and educational facilities in Ghana, encompassing both urban and rural settings, to ensure a representative sample of the respiratory therapy profession's current status in the country. Participants were primarily healthcare professionals, including respiratory therapists (RTs), RT students, faculty members, physicians, nurses, and midwives. We aimed to gather a comprehensive view of the profession from those directly and indirectly involved in RT services.

Inclusion criteria were RTs, RT students, faculty, physicians, nurses, and midwives able to read, write, and speak English. Exclusion criteria included medical students and nurses/midwives currently enrolled in educational programs, as well as individuals unable to read, write, and speak English. A survey link was sent to members of the following associations: Ghana Medical Association, Ghana Registered Nurses and Midwives Association, Christian Health Association of Ghana (CHAG), Ghana Association for Respiratory Care, and Respiratory Department of the School of Allied Health Professional, University of Ghana, Legon. The snowball sampling method was used as well. The subjects’ informed consent was embedded in the survey as an option that must be agreed to in order to proceed with the survey.

Survey questions

Subjects were asked to respond to an anonymous survey questionnaire about the field of respiratory therapy in Ghana. Questions included demographics, needs assessment-related questions, the respiratory therapy profession, knowledge of respiratory diseases, advantages, challenges, and barriers to the profession in Ghana. Other questions asked were knowledge of respiratory equipment, the workload of a respiratory therapist, qualifications, and years of experience. In addition, loyalty and recommendations of the profession were assessed using the Net Promotor Score (NPS) using a validated 0-10 scale question if they would refer the profession to a friend or a colleague [21-23]. The survey included the following types of questions: multiple-choice questions, open-ended questions, Likert scale questions, rating scale questions, and yes/no questions, which are described in the results section. The data was collected using the Qualtrics survey application.

Analysis

Statistical analyses were conducted using IBM Corp. Released 2023. IBM SPSS Statistics for Windows, Version 29.0.2.0 Armonk, NY: IBM Corp. Data were summarized using descriptive statistics: frequencies and percentages for categorical variables, mean ± standard deviation (SD) for quantitative variables.

## Results

Initially, 201 individuals responded to the survey invitation, with 195 agreeing to take the survey (Table 1), which represents the demographic characteristics of the study population. The demographic breakdown of the participants showed a gender distribution of 83 males (42.6%) and 112 females (57.4%). Most participants were in the 30-39 age group, representing 51.1% of the sample, with a mean age of 32.33 years (SD ± 8.83). The majority of participants (56.4%) held bachelor's degrees, followed by diplomas and doctorates (12.8%) each. When asked about their professional background, the majority were nurses (35.9%), physicians (21.9%), and respiratory therapists (7.3%).

**Table 1:** Demographic analysis of participants in the survey on respiratory therapy in Ghana *n= represents the number of participants who answered the demographic questions. The data has been represented as N and %.

Variables	Values	n*	%
Gender			
	Male	83	42.6
	Female	112	57.4
Age	17 -19	9	4.8
	20 – 29	57	30.3
	30 – 39	96	51.1
	40 – 49	19	10.1
	50 and above	7	3.7
Education			
	Diploma [associate degree]	25	12.8
	Bachelor’s degree	110	56.4
	Master’s Degree	25	12.8
	Doctorate [MD/PhD]	23	11.8
	Other [Certificate, Senior high score, Fellowship Post graduate surgery]	12	6.2
Profession program			
	Physician	42	21.9
	Nurse	69	35.9
	Respiratory Therapist	14	7.3
	Respiratory Care Student	21	10.9
	Midwife	20	10.4
	Other [Radiographer, Public health]	26	13.5
Geographical Regions in Ghana			
	Greater Accra	83	43.2
	Western	23	12
	Ashanti	22	11.5
	Northern	21	10.9
	Central	10	5.2
	Volta	8	4.2
	Bono East	6	3.1
	Eastern	5	2.2
	Upper East	4	2.1
	Ahafo	2	1
	Bono	2	1
	Upper West	2	1
	Savannah	1	0.5
	Northeast	1	0.5
	Oti	0	0
Physicians' Roles and Responsibilities			
	Research	14	35.9
	Ministry work	2	5.1
	Patient care	39	100
	Administrative work	13	33.3
	Other	3	7.7
Years of Experience in the current profession?			
	1 to 5	53	43.4
	6 to 10	32	26.2
	11 to 15	22	18.0
	16 to 20	11	9.0
	21 +	4	3.3

**Figure 1:**
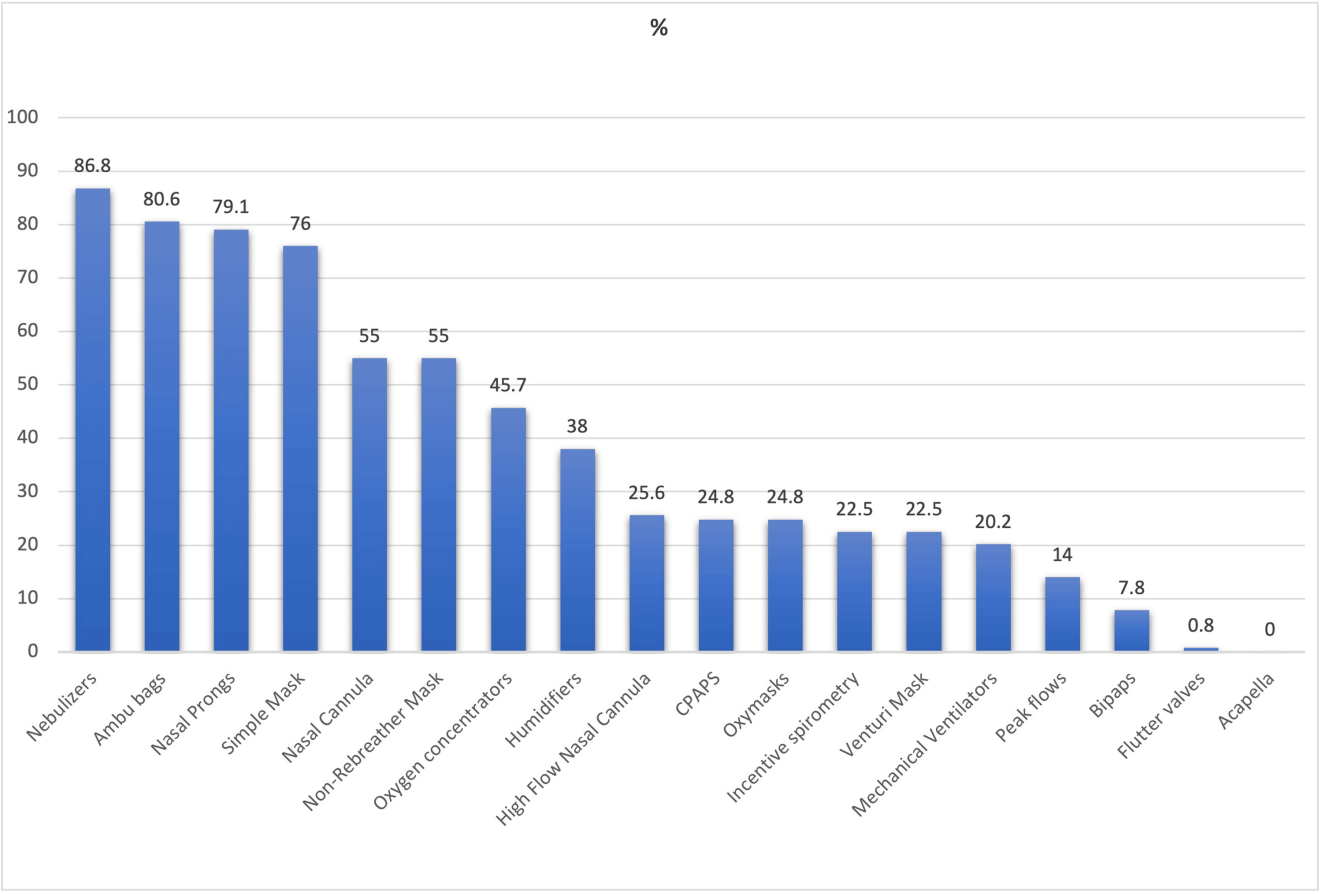
Familiarity with respiratory-related devices and equipment Data is represented as percent (%) of total N.

Table 2 represents the evaluation of the needs for respiratory therapists in Ghana with 129 individuals who completed the survey. Insights from the survey indicated a critical need for more respiratory therapists, as 64.2% of participants endorsed increasing the number of practitioners. However, the interaction with RTs was minimal; 73.6% of respondents reported they had never worked with a respiratory therapist, which points to a significant gap in the current healthcare system. Figure 1 shows the majority of the participants were familiar with nebulizers (86.8%) and Ambu bags (80.6%), with 75% of participants preferring to start a respiratory therapy major early at the bachelor's level. 

**Table 2:** Analysis of the needs for respiratory therapy in Ghana *n= Only students and faculty responded to these questions. The data has been represented as N and %

Variables	Values	n*	%
Support For Increasing the Number of RTs In Ghana	Yes	129	64.2
	No	0	0
Interact or worked with RTs before	Yes	34	26.4
	No	95	73.6
Preferred Educational Level for Entry to The Profession Degree			
	Bachelor	93	75.0
	Masters	13	10.5
	Doctorate	6	4.8
	Other [The West African Senior School Certificate Examination, Diploma]	12	9.7
Recommendations for Pursuing a Career as a Respiratory Therapist			
	Yes	119	96.0
	No	5	4.0

Table 3 illustrates the evaluation of the current state of respiratory therapy education and practice in Ghana. Regarding the current year or level in the respiratory therapy program, the study findings show a distribution of students across different levels: 15.0% in Level 100, 45.0% in Level 200, 25.0% in Level 300, and 15.0% in Level 400.

**Table 3:** Evaluating for the current respiratory therapy students and practicing respiratory therapists in Ghana respiratory program. Total sample size (n) is 20. **Table 2 presents workload classifications as reported by respiratory therapists in Ghana, with a sample size of 14. The data has been represented as N, %

		n*	%
Variables	Values		
Current Year or Level in the Respiratory Program*(n=20)			
	Level 100	3	15.0
	Level 200	9	45.0
	Level 300	5	25.0
	Level 400	3	15.0
Classification of Workload for Respiratory Therapists in Ghana (n=14)- **			
	Overwhelming	9	64.3
	Normal	5	35.7
	Below normal	0	0
Major Challenges in the Respiratory Therapy Profession in Ghana (n= multiple answer)			
	Lack of or insufficient recognition of the value of the respiratory care profession	14	100
	Shortage of RT professionals	11	78.6
	Lack of awareness	13	92.9
	Limited resources	12	85.7
	Lack of standardized training program	10	71.4
	Low salary	11	78.6
	Lack of or limited research opportunities	10	71.4
	Other	1	7.1

The workload for respiratory therapists was considered overwhelming by 64.3% of respondents, highlighting the stressful conditions under which these professionals operate. Major challenges faced by the profession included a complete lack of recognition noted by all respondents (100%), resource shortages (85.7%), and the absence of standardized training programs (71.4%).

**Figure 2:**
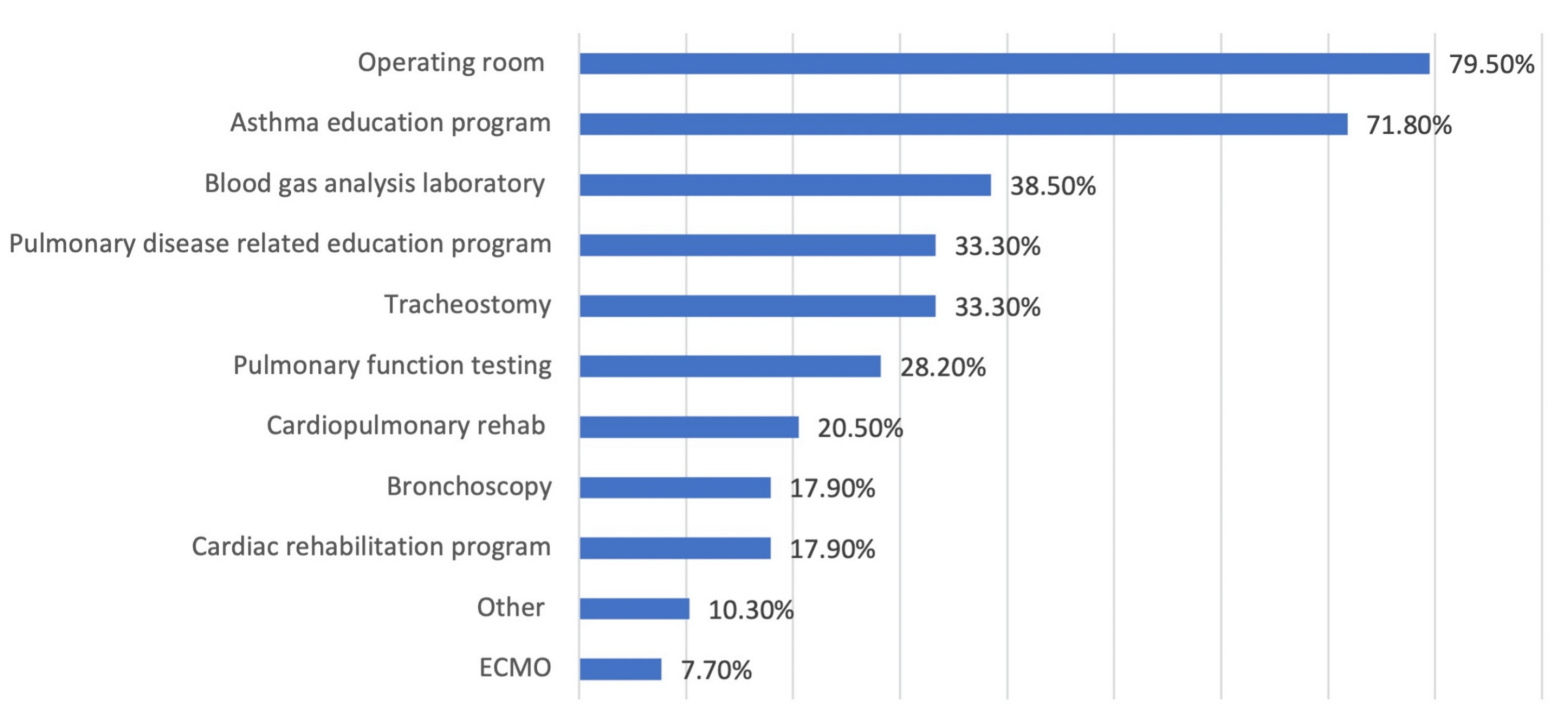
The availability of cardiopulmonary services in hospitals The data has been represented as percent (%)

**Figure 3:**
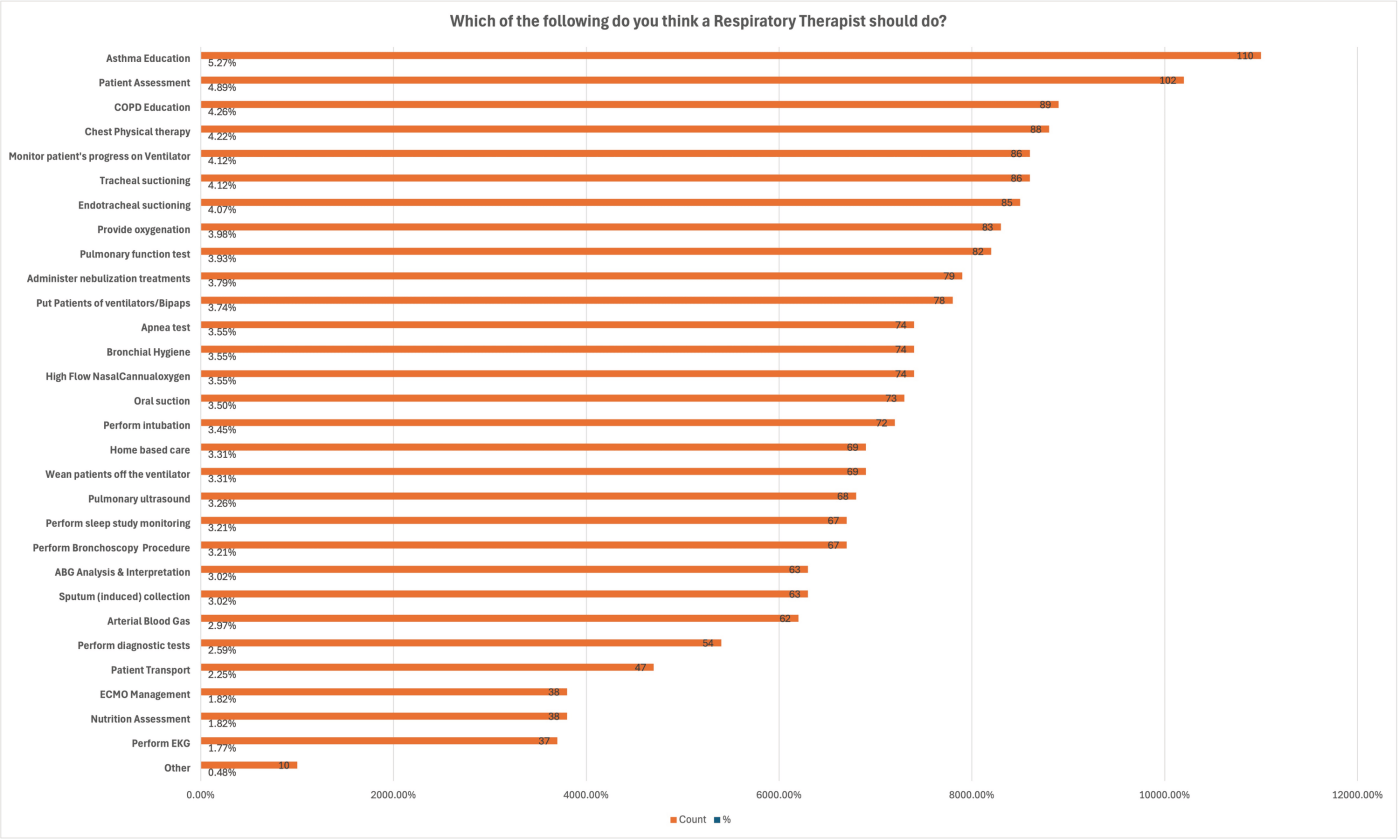
Recommended responsibilities for respiratory therapists This question was a multiselect question where each subject selected as many areas as they believe it should be part of the responsibility for a respiratory therapist. Data is expressed as count and percent and organized accordingly in this figure. For example, asthma shows education showed the highest selection that was received among participants, followed by patient assessment and COPD.

Educational demographics revealed a balanced distribution across academic years, with a slight majority (45.0%) in their second year. Moreover, Figure 2 indicated that operating rooms (79.5%), asthma education programs (71.8%), and blood gas analysis laboratories (38.5%) are the three most accessible services. Interestingly, Figure 3 shows that asthma education is the top-rated responsibility for respiratory therapists at 88.7%, while nutrition assessment and ECMO management are among the least emphasized at 30.6%, indicating specialized roles within the RT's scope of practice.

As illustrated in Table 4, the current number of respiratory-related supplies in Ghana, the study observed a varied distribution of resources across different healthcare facilities, with 93% of facilities reporting having no RTs, with only a few facilities having between one and four therapists. Regarding facility resources, 36.3% of healthcare facilities have fewer than 50 beds, while an equal percentage have more than 200 beds. Non-invasive mechanical ventilators are available in 40.1% of these facilities; however, inventory uncertainties are high, with 47.3% unable to provide precise figures. The number of ICU beds is 6.98 ± 12.50, indicating significant variability in bed availability. This issue of uncertainty is more pronounced for invasive ventilators, with 56.0% of facilities reporting uncertainty in providing exact numbers.

**Table 4:** The current number of resources related to respiratory in Ghana based on the respondents own facilities *missing data from respondents. The data has been represented as N, %

Variables	Values	n*	%
Total Number of hospital beds in			
	1 to 49	45	36.3
	50 to 99	21	16.9
	100 to 199	13	10.5
	200 +	44	36.3
Number of Non-Invasive Mechanical Ventilators			
	1 to 5	73	40.1
	6 to 10	15	8.2
	11 to 20	7	3.8
	21 to 30	0	0
	30+	1	.5
	I do not know	86	47.3
Number of Invasive Mechanical Ventilators			
	1 to 5	52	28.6
	6 to 10	17	9.3
	11 to 20	6	3.3
	21 to 30	4	2.2
	31 +	1	.5
	I do not know.	102	56.0
Number of Respiratory Therapist at the facility			
	1	12	6.6
	2	10	5.5
	3	9	4.9
	4+	12	6.6
	None	99	54.4
	I do not know	40	22.0

Finally, the Net Promoter Score (NPS) shows that 49% of the sample was promotor, 28% was passive, and 23% was detractor. The overall NPS score was 26.92, indicating moderate loyalty to the respiratory therapy profession within Ghana.

## Discussion

The objective of this study was to assess the status of the RT profession in Ghana. To our knowledge, this is the first study that evaluated the status of the RT profession in the country.

Our findings show the need for RTs and the inadequacy of respiratory support when it comes to devices and support systems. For example, as shown in Figure 2, the majority of the respondents are comfortable using only low-flow systems such as nasal cannulas, masks, nebulizers, etc. On the contrary, very few are comfortable with using high-flow devices such as invasive devices (ventilators). This was supported by the low ventilator counts at their facilities. This is consistent with the literature which shows the inadequacy of supplies and support where policymakers and developed countries should consider investing in the infrastructure to support the growing RT profession [5]. Furthermore, studies have reported how invasive devices such as mechanical ventilators are challenging and not always available, whereas non-invasive modalities have been used instead, which our findings support. Also, the low familiarity of Bipaps (7.8%) is an indication of inadequate support for ventilatory patients [24,25].

Furthermore, among the respondents, the results show very low RT numbers within the facilities, which suggests that most likely the duties are handled by other professionals who are not specialized in respiratory therapy. The low RT count is a challenge not just in Ghana but also worldwide. In the United States, it has been reported that the professional organization is calling for more RTs and has increased efforts to recruit more students to be involved in the profession due to various factors such as COVID-19, the retirement of many RTs in the profession, and burnout in general. [16,26-28] We thus believe that Ghana is moving in the right direction by creating a program within the country, and thus more programs will hopefully support closing the gap and need for treating cardiopulmonary diseases by having more RTs in the workforce.

When it comes to the education level of the profession, the majority of the respondents supported having the profession be at a minimum level of baccalaureate and higher. This is following the profession movement worldwide by the American Association of Respiratory Care (AARC) and Coalition for Bachelors and Graduate Respiratory Therapy Education. The movement has been shifted to support the need for a minimum bachelor’s degree as it is linked to having a higher understanding of evidence-based medicine as well as improving patient care [28,29].

The support from the respondents regarding the profession is seen clearly when viewing the results of the NPS scores. The majority support having a RT profession by showing a high promotor score and being passive. To our knowledge, this is the first study that utilized this validated scale that is typically used in business and some healthcare settings to show loyalty [21,22].

Strengths of the study

We believe that this study has several strengths. First, it is the first needs assessment that evaluated the status of the RT profession in Ghana. Thus, the data and results can be used by policymakers to shape the future of the profession. Second, education programs can use the findings to shape their curriculum in terms of what stakeholders, such as physicians, look for. This study benefits from several key strengths that enhance its contribution to the literature on respiratory therapy in Ghana. Firstly, the comprehensive participant representation from various professional backgrounds (respiratory therapists, physicians, nurses, and midwives) ensures diverse perspectives and enhances the generalizability of the findings. Secondly, the robust methodological framework, employing both quantitative and qualitative data collection methods, provides a comprehensive dataset for analysis. The innovative use of the Net Promoter Score (NPS) is particularly notable as it introduces a business metric to health services research in Africa, providing new insights into professional satisfaction and advocacy within the healthcare sector.

Limitations of the study

This study has some limitations. First, the sample size might be considered low for the country. However, the authors have worked on increasing the efforts to recruit more samples in terms of physicians and other healthcare providers. In addition, a challenge was reaching out to different hospital sites. Future studies should consider a prospective study approach to assess the effectiveness of the current RT workforce in improving patient outcomes. The study's scope primarily included one institution, which may limit the generalizability of the findings across Ghana. Future studies should aim to include a broader range of institutions to validate these findings. The sampling method, snowball sampling, although necessary for reaching a targeted population, might introduce bias as it does not guarantee a representative sample of all healthcare professionals in Ghana.

## Conclusions

The results of this study show the need and support for having more RTs in Ghana. Various key stakeholders are showing strong support for having more RTs. We hope that the findings of this study assist key policymakers in the country in shaping the future of healthcare in Ghana, where respiratory therapists play a major role in improving patient care outcomes in cardiopulmonary disease.
